# Interfacial Characterization of Polypyrrole/AuNP Composites towards Electrocatalysis of Ascorbic Acid Oxidation

**DOI:** 10.3390/molecules27185776

**Published:** 2022-09-07

**Authors:** Camila Pesqueira, Bruna M. Hryniewicz, Larissa Bach-Toledo, Luciane Novaes Tenório, Luís F. Marchesi, Talita Mazon, Marcio Vidotti

**Affiliations:** 1 Grupo de Pesquisa em Macromoléculas e Interfaces, Departamento de Química, Universidade Federal do Paraná (UFPR), Curitiba 81531-980, PR, Brazil; 2 Centro de Tecnologia da Informação Renato Archer (CTI), Rod. D. Pedro I, KM143.6, Campinas 13069-901, SP, Brazil; 3 Grupo de Estudos em Espectroscopia de Impedância Eletroquímica (GEIS), Universidade Tecnológica Federal do Paraná, Av. Monteiro Lobato, s/n–Jardim Carvalho, Ponta Grossa 84017-220, PR, Brazil

**Keywords:** electrocatalysis, gold nanoparticles, conducting polymers, overoxidation, electrochemical impedance spectroscopy

## Abstract

Polypyrrole (PPy) is an interesting conducting polymer due to its good environmental stability, high conductivity, and biocompatibility. The association between PPy and metallic nanoparticles has been widely studied since it enhances electrochemical properties. In this context, gold ions are reduced to gold nanoparticles (AuNPs) directly on the polymer surface as PPy can be oxidized to an overoxidized state. This work proposes the PPy electrochemical synthesis followed by the direct reduction of gold on its surface in a fast reaction. The modified electrodes were characterized by electronic microscopic and infrared spectroscopy. The effect of reduction time on the electrochemical properties was evaluated by the electrocatalytic properties of the obtained material from the oxidation of ascorbic acid (AA) and electrochemical impedance spectroscopy studies. The presence of AuNPs improved the AA electrocatalysis by reducing oxidation potential and lowering charge transfer resistance. EIS data were fitted using a transmission line model. The results indicated an increase in the electronic transport of the polymeric film in the presence of AuNPs. However, PPy overoxidation occurs when the AuNPs’ deposition is higher than 30 s. In PPy/AuNPs 15 s, smaller and less agglomerated particles were formed with fewer PPy overoxidized, confirming the observed electrocatalytic behavior.

## 1. Introduction

Unlike conventional polymers, conductive polymers are organic structures with electrical and optical properties, similar to inorganic semiconductors [1]. The presence of conjugated bonds in the chain is responsible for these properties since electrons π can be easily removed for the polymer doping, and the electronic delocalization creates a path for the charge mobility [1,2,3]. Due to their well-reported properties, conducting polymers are applied in many areas [4,5,6,7,8]. Among these properties, the ability to catalyze some electrode reactions, changing the electrochemical potential, motivates the study of these materials for different applications such as fuel cells and sensors [9,10,11].

Among the conducting polymers, polypyrrole (PPy) is particularly interesting since it exhibits high conductivity, good environmental stability, and biocompatibility [1,12]. Moreover, the association between conducting polymers and metal nanoparticles has been widely studied, on promoting, enhancing, and exploring their properties. There are many methods to assemble these composites, and several lead to sophisticated techniques and usually consist of multistep processes [13,14]. However, some studies proposed the direct reduction of Au on polymer structures without any reducing agent, eliminating possible contamination and cutting off steps [15,16]. It is assumed that PPy is oxidized to an overoxidized state by HAuCl_4,_ and the Au^3+^ ions are reduced to AuNPs on the polymer surface. As a result, the conductivity of the overoxidized PPy composite decreases, even with partial overoxidation, since an insulating layer of overoxidized PPy [15] separates the metallic nanoparticles and the conducting polymer. In this context, we propose the PPy electrochemical synthesis followed by the direct reduction of gold on its surface in a fast reaction. Additionally, electrocatalysis assays can be employed to study and exploit the properties of the new materials.

Based on this, ascorbic acid (AA) was selected as a model molecule to perform and evaluate its electrocatalysis with the synthesized composites. The L enantiomer of ascorbic acid, also called vitamin C, plays an essential role in biological activities and is found naturally in vegetables and fruits. The AA can perform as a hydrophilic antioxidant since it is easily oxidized to dehydroascorbic acid, protecting cell components against free radicals formed during metabolism. Controversially, in some conditions, AA can also present a pro-oxidative behavior and affect the activity of the cells [17,18]. Due to its reductive properties, AA is primarily used as an antioxidant in foods, drinks, and pharmaceutical formulations preventing unwanted color or flavor changes [19,20,21,22]. AA is also known for its large oxidation overpotential on conventional electrodes, and new composite materials have been reported in the literature for its determination from electrocatalysis [23,24,25,26,27,28,29].

An excellent way to evaluate the changes in the electrochemical properties in the presence of AuNPs is by using electrochemical impedance spectroscopy (EIS), which is a powerful tool for understanding the electrode/electrolyte interface characteristics providing information about the electrocatalytic features of the modified electrodes. In this context, the present work provides a detailed study of the influence of AuNPs directly reduced on PPy film in the electrocatalytic properties of the obtained material through EIS experiments using AA as a probe.

## 2. Results and Discussion

**Figure 1:**
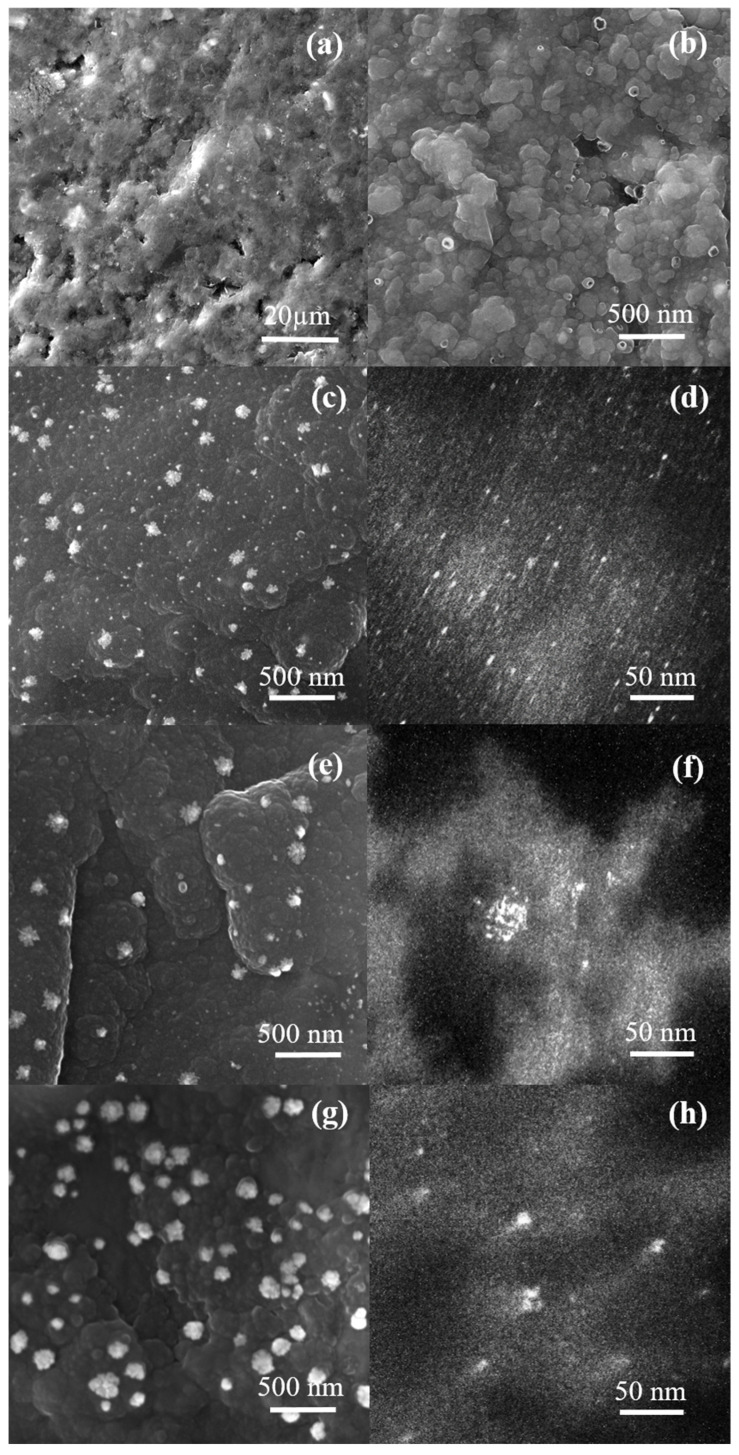
Representative SEM and dark-field TEM images, respectively, of bare graphite (**a**), modified graphite electrodes with PPy (**b**), PPy/AuNPs 15 s (**c**,**d**), PPy/AuNPs 30 s (**e**,**f**) and PPy/AuNPs 45 s (**g**,**h**).

The surface morphologies of the bare pencil graphite electrode and the modified electrodes were characterized by SEM and TEM images, as shown in Figure 1 and Figure S1. The unmodified electrode (Figure 1a) showed a porous structure seen elsewhere [30,31]. On the other hand, for PPy (Figure 1b), a characteristic globular morphology was obtained [14,31], and, even after the modification with AuNPs, the globular morphology was maintained, as observed in Figure 1c,e,g and Figure S1. Furthermore, the presence of AuNPs was observed on the PPy surface (Figure 1c,e,g), and similar results were observed previously [14]. Moreover, it is possible to notice that in the PPy/AuNPs 5 s (Figure S1) and PPy/AuNPs 15 s images (Figure 1c), the formation of smaller and less agglomerated particles are observed than in the 30 s and 45 s images (Figure 1e,g). Through dark-field TEM images of PPy/AuNPs 15 s, small gold nanoparticles can be observed all over the PPy surface (Figure 1d), while for PPy/AuNPs 30 s and 45 s, more oversized agglomerates are observed (Figure 1f,h).

**Figure 2:**
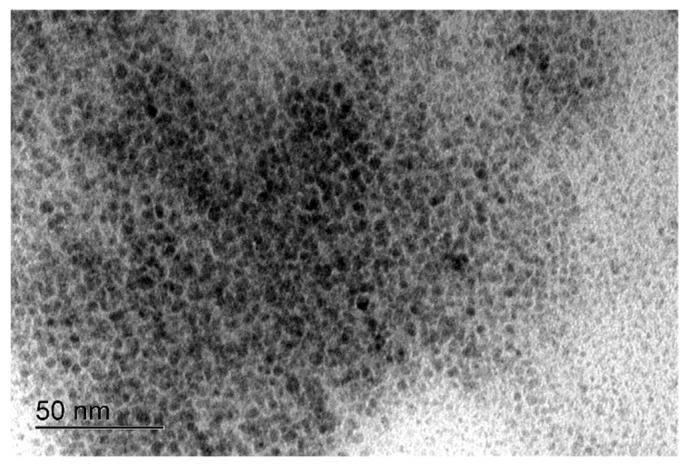
Representative bright-field TEM image of PPy/AuNPs 15 s.

In the bright-field image of PPy/AuNPs 15 s (Figure 2), the small gold nanoparticles are seen, with a mean size of 3.7 ± 0.9 nm, while for the 30 s and 45 s materials, they are not apparent (Figure S2a,b). In this way, the size of the nanoparticles could not be obtained.

**Figure 3:**
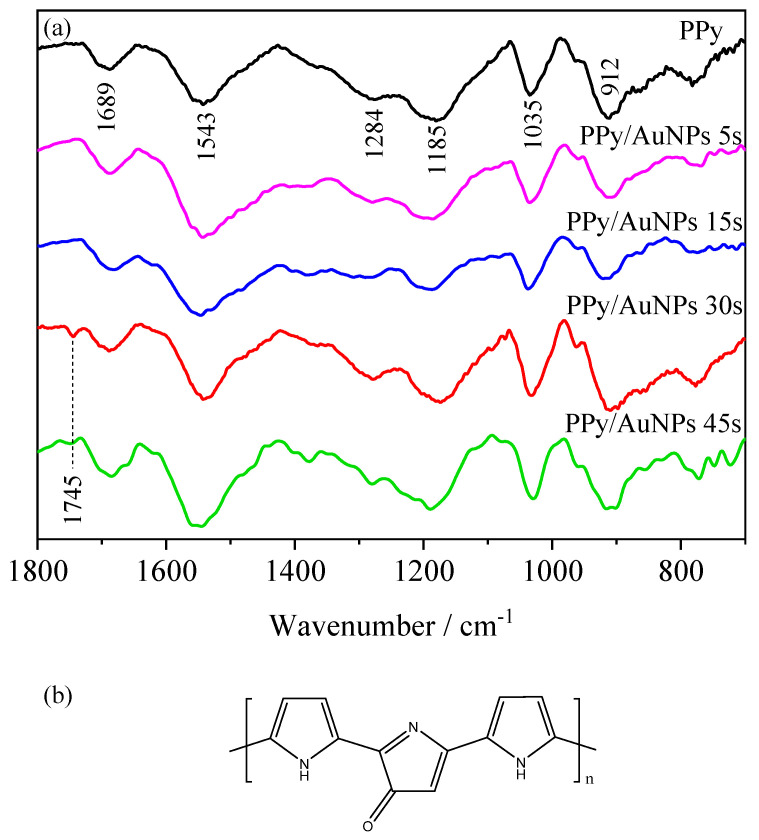
(**a**) FTIR spectra of PPy (black line), PPy/AuNPs 5 s (pink line), PPy/AuNPs 15 s (red line), PPy/AuNPs 30 s (blue line) and PPy/AuNPs 45 s (green line) modified graphite electrodes. (**b**) Structure of overoxidized PPy [32,33].

The PPy and PPy/AuNP materials were characterized by FTIR spectra, as shown in Figure 3a.

The characteristic PPy bands are seen in all ranges, as in 912 cm^−1^ related to the breathing of pyrrole and in-plane C-H deformation, in 1035 cm^−1^ assigned to C-C out of plane deformation [34], in 1185 cm^−1^, indicating the doping state of PPy [35], in 1284 cm^−1^ corresponding to C-H and C-N in-plane deformation mode [36] and 1543 cm^−1^, related to pyrrole ring stretches [37]. In the PPy/AuNPs 30 s and 45 s spectra, a band at 1745 cm^−1^ appeared, associated with the C = O vibrations formed due to the overoxidation of PPy at the β-C pyrrole ring [15,33]. The structure of overoxidized PPy can be found in Figure 3b [32,33]. Nevertheless, this band did not appear in the pure PPy and PPy/AuNPs 5 s and 15 s spectra, indicating that, in these cases, PPy was not overoxidized.

**Figure 4:**
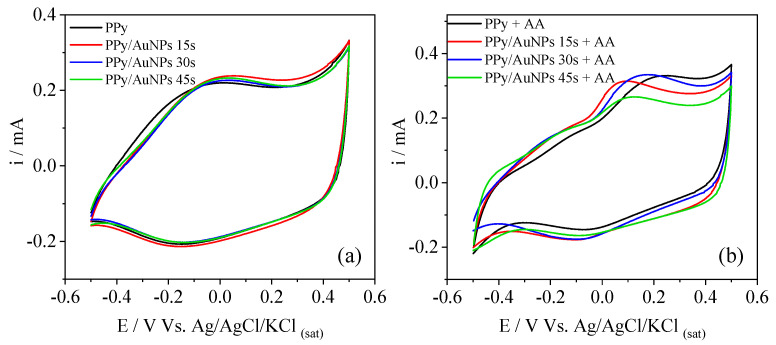
Cyclic voltammetry of PPy, PPy/AuNPs 15 s, PPy/AuNPs 30 s, and PPy/AuNPs 45 s in (**a**) PBS electrolyte and (**b**) PBS electrolyte containing 5 mmol L^−1^ of AA. Scan rate = 20 mV s^−1^.

For shorter times, the gold ions’ reduction is balanced with the PPy oxidation from neutral to polaronic and bipolaronic states of the chain, and to the overoxidized states in the highly oxidized region. In this case, the overoxidation degree is probably very low and the bands for the overoxidized PPy cannot be seen in the FTIR results. On the other hand, for 30 s and 45 s, oxidation occurs for longer times, reaching higher PPy overoxidized states. In order to verify the influence of the AuNPs on the electrochemical behavior of PPy, the materials were characterized by cyclic voltammetry in PBS (Figure 4a)

The materials were electrochemically characterized by cyclic voltammetry in PBS to verify the influence of the AuNPs on the electrochemical behavior of PPy (Figure 4a). The voltammograms show the typical PPy profile, with the redox processes and a high capacitive current. However, the presence of AuNPs did not affect the PPy voltammetric behavior greatly. In this way, AA was inserted into the electrolyte to better understand the differences in the electrochemical behavior in the presence of AuNPs, such as the electrocatalytic properties (Figure 4b).

In the presence of AA, an oxidation process at 0.2 V appeared for PPy, related to the oxidation of AA to dehydroascorbic acid [20,21,23]. This potential value is similar to others obtained elsewhere [27,38,39]. As reported in the literature, an electroinactive product can be formed when L-ascorbic acid oxidation occurs. Herein the dehydro-L-ascorbic acid opens its lactone ring, leading to by-products that quickly adsorb onto electrode surfaces. Consequently, electrode fouling is reported [19,22,40,41,42]. The problem with electrode fouling is the significant difficulty in determining AA by electrochemical measurements. In our case, despite AA being selected as a probe and no determination of it being proposed, SEM images were taken to verify the electrode morphology after the CV measurements. Through SEM images (Figure S3) after CV, neither PPy globular morphology is evidenced, nor do the AuNPs appear (as previously observed in Figure 1a), suggesting the presence of a fouling and non-electroactive material recovering the surface. In addition to that, from FTIR of PPy/AuNPs 15 s after AA catalysis (Figure S4), a band at 1090 cm^−1^ appeared, which may be related to the C-OH vibration of the by-products formed during the process [43].

**Figure 5:**
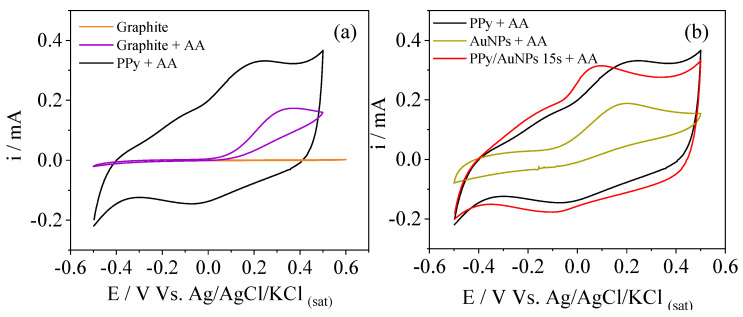
(**a**) Cyclic voltammetry of pencil graphite electrode in PBS electrolyte (orange line), bare pencil graphite (purple line), and PPy (black line). (**b**) Cyclic voltammetry of PPy (black line), AuNPs (yellow line), and PPy/AuNPs 15 s (red line) in PBS electrolyte containing 5 mmol L^−1^ of AA. Scan rate = 20 mV s^−1^.

Electrocatalytic oxidation of AA at PPy compared to the bare graphite electrode was demonstrated, as shown in Figure 5. This potential value is similar to others obtained elsewhere [27,38,39]. In the presence of AuNPs (Figure 4b), this process occurred at lower energetic potentials, 0.05 V for AuNPs 15 s, 0.13 V for AuNPs 30 s, and 0.07 V for AuNPs 45 s, indicating an improvement in AA electrocatalysis by the metallic nanoparticles. The lower energetic potential for AuNPs 15 s could be related to the higher AuNPs’ surface area in this sample since their size is smaller. In addition, their good distribution on the PPy surface and the short time used to reduce the gold ions avoid an overoxidized state of PPy. The presence of AuNPs in the porous PPy film reduces the resistance by facilitating the electron transfer, enhancing the AA electrocatalysis [44,45]. PPy/AuNPs 5 s electrode does not present reliable results to discuss, probably due to the short synthesis time of the gold nanoparticles, which makes slight variations in the time strongly affect the electrocatalysis and, consequently, the reproducibility.

The comparisons between the electrocatalysis of AA, performed by the bare graphite electrode and modified electrodes with PPy are presented in Figure 5a, where the electrocatalytic effect of PPy on AA oxidation can be clearly seen. CVs of AA oxidation performed by PPy, electrochemically reduced AuNPs (for comparison), and PPy/AuNPs 15 s are shown in Figure 5b. Both AuNP- and PPy-modified pencil graphite electrodes show a electrocatalytic behavior for AA oxidation when compared to the bare graphite electrode. However, for the PPy/AuNPs 15 s modified electrode a synergistic effect can be observed and the oxidation potential of the reaction is decreased.

**Figure 6:**
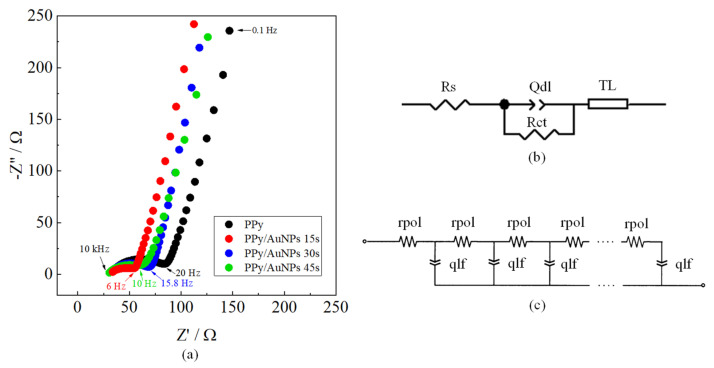
(**a**) Nyquist diagrams of PPy, PPy/AuNPs 15 s, PPy/AuNPs 30 s, and PPy/AuNPs 45 s in PBS containing 5 mmol L^−1^ of AA at a dc potential of 0.4 V. (**b**) Equivalent circuit used to fit EIS data, and (**c**) the transmission line (TL) model is presented in the equivalent circuit.

To better understand the influence of the AuNPs on the PPy electrochemical behavior, the modified electrodes were also characterized by electrochemical impedance spectroscopy (EIS) measurements in the presence of the AA as a probe (Figure 6a), applying a dc potential of 0.4 V, which is sufficient to oxidize AA in all materials. The equivalent circuit used to fit the EIS data is shown in Figure 6b. The semicircle was fitted using: a series resistance (*Rs*), accounting for the resistance of the electrolyte, cables, and current collectors; a charge transfer resistance (*Rct*), related to the facility of transferring the charges at the electrode/electrolyte interface; and a constant phase element (CPE) related to the double-layer capacitance at the electrode/electrolyte interface (*Qdl*), with an *ndl* varying from 0 to 1, where the unity represents an ideal capacitor.

The 45° straight line in the high-frequency region and the line approaching 90° in the low-frequency region were fitted using a transmission line model (TL), where a potential drop inside the polymeric film is considered, which is typical for the polymeric film due to the porous nature (Figure 6c) [46]. This model includes an χ distributed element, which is an impedance per unit length (Ω m^−1^), transverse to the macroscopic outer surface, representing the potential drop at the conducting polymer film, and a ζ distributed element, which is an impedance length (Ω m), parallel to the macroscopic surface, where the charge accumulation in the polymeric film to maintain charge neutrality upon the oxidation process is considered. The χ distributed element was substituted by a resistance (*rpol*) related to the electronic transport in the polymeric film [47], and the ζ distributed element was replaced by a constant phase element, related to the charge accumulation process inside in/out the polymeric film to maintain the electroneutrality during the redox processes (*qlf*), with an ideality factor (*ndl*), related to the homogeneity of the intercalation process [48]. The parameters obtained through fitting EIS data are shown in Table 1, and the fitting errors are presented in Table S1.

*Rs* variations cannot be adequately discussed due to changes in the cell constant at each experiment. *Rct* values, which are related to the resistance to oxidizing AA at the surface of the material, decreased in the presence of AuNPs, especially for the AuNPs 15 s, highlighting the electrocatalytic behavior of the metallic nanoparticles, as was also verified in the cyclic voltammetry, showed previously (Figure 4). As well as their electrocatalytic behavior, the size, distribution, and quantity of the AuNPs may also influence the oxidation process of the AA. The double-layer capacitance, represented by *Qdl*, increased for PPy/AuNPs 15 s compared to bare PPy, indicating a higher electroactive area for this material, followed by a decrease in the *ndl*, showing a less homogeneous surface in the presence of the AuNPs. We can see a more heterogeneous AuNP distribution for this sample (Figure 1a,b). However, for PPy/AuNPs 30 s and PPy/AuNPs 45 s, the *ndl* parameter almost did not change in comparison with PPy, probably due to the high number of AuNP aggregates formed in this case, as seen in the SEM images (previously shown in Figure 1c,e). The *rpol* parameter evidences a more conducting form of PPy in the presence of AuNPs, mainly for PPy/AuNPs 15 s, indicating that the AuNPs favor a higher doping state of the polymer. This was verified before for a poly (3,4-ethyelenedioxythiophene) (PEDOT) and AuNP composite [49]. For PPy/AuNPs 30 s and PPy/AuNPs 45 s, *rpol* increased compared to PPy/AuNPs 15 s, probably due to the PPy overoxidation caused by the AuNPs’ formation, as seen in the FTIR spectra. PPy *qlf* and *nlf* parameters almost did not change in the presence of AuNPs, showing that the ionic intercalation process is not affected by the presence of the nanoparticles; similar results were previously observed [49].

## 3. Materials and Methods

### 3.1. Chemicals and Materials

All solutions were prepared with ultrapure water (Elga system, R = 18.2 MΩ·cm). Pyrrole monomer (98% from Sigma-Aldrich) was purified by distillation under low pressure and kept in a refrigerator (−18 °C). The following reagents were analytical grade and used as received: Na_2_HPO_4_, KCl, HAuCl _4_·3H_2_O (30% wt in HCl) were purchased from Sigma-Aldrich; KH_2_PO_4_, NaCl, HNO_3_ from Synth; L-ascorbic acid (AA) from Biotec; and KNO_3_ from Merck. The electrochemical measurements were conducted in IviumStat potentiostat using a pencil graphite electrode (2 mm in diameter and area of 0.47 cm^2^) as the working electrode, a platinum foil as the counter electrode, and Ag/AgCl/Cl^−^ (3M) as the reference electrode.

### 3.2. Electrochemical Synthesis and Gold Reduction

To perform the globular polypyrrole (PPy) synthesis, a conventional three-electrode cell was designed, and the methodology was based on a previous work of our research group [50]. The electrochemical medium constitutes 50 mmol L^−1^ of the monomer pyrrole and 8 mmol L^−1^ KNO_3_, and the pH of the solution was adjusted to pH 2 with HNO_3_ aliquot (1 mol L^−1^). The electropolymerization was carried out under potentiostatic conditions, applying 0.8 V and with charge control of 300 mC cm^−2^, to ensure the amount of polymer in the working electrode was kept the same between the syntheses.

The gold reduction was made directly on the working electrode surface. After the PPy synthesis, the modified electrode was washed with deionized water and immersed in an aqueous solution of HAuCl_4_ (0.25 mmol L^−1^) at different times of 5, 15, 30, and 45 s.

As matter of comparison, a pencil graphite electrode was modified with AuNPs by electrochemical reduction at −1.1 V for 15 s in a solution containing 0.25 mmol L^−1^ HAuCl_4_ and 0.1 mol L^−1^ KCl.

### 3.3. Characterization

All the electrochemical characterizations were made in 0.1 mol L^−1^ phosphate buffer saline (PBS) at pH 7.4 in a three-electrode cell. Initially, the electrodes were characterized by cyclic voltammetry (CV) in a potential range from −0.5 V to 0.5 V. The ascorbic acid electrocatalysis was performed in PBS containing 5 mmol L^−1^ of ascorbic acid. The EIS measurements were made at a dc potential of 0.4 V and ac potential of 10 mV, with the frequency ranging from 10 kHz to 100 mHz. The morphologies of the modified electrodes were investigated by Scanning Electron Microscopy (SEM) in a TESCAN MIRA3 (Kohoutovice, Czech Republic) and Transmission Electron Microscopy (TEM) images in a JEOL JEM 1200EX-II (Tokyo, Japan). Attenuated total reflection Fourier transform infrared (ATR-FTIR) spectra were collected in a Thermo Scientific Smart iTR Nicolet iS10 (Madison, WI, USA).

## 4. Conclusions

PPy was electrochemically synthesized onto a pencil graphite electrode, and AuNPs were successfully reduced on the PPy surface by a short-time reaction. The modified electrodes were characterized by microscopic, electrochemical, and spectroscopic techniques. As a result, a relation between the time of Au reduction exposure and the improvement in the electrocatalytic properties was observed. Exposure to the gold ion solution at only 15 s can reduce the AuNPs with less PPy overoxidized, enhancing the electrocatalytic properties. Although this relation has been observed in cyclic voltammetry, EIS analysis was essential to explore and understand the electrolyte/electrolyte changes in the presence of AuNPs synthesized with different reduction times. The results indicate that the hybrid material of PPy/AuNPs has a great potential to be used in further sensor applications based on electrocatalytic reactions.

## Figures and Tables

**Table 1:** Parameters obtained by fitting EIS data are shown in Figure 6a.

Electrode	Rs/Ω	Rct/Ω	Qdl/mF s^n−1^	Ndl	Rpol/Ω	Qlf/mF s^n−1^	Nlf
PPy	31.73	47.93	0.170	0.64	23.98	6.09	0.827
PPy/AuNPs 15 s	30.69	24.38	0.816	0.514	8.176	6.00	0.848
PPy/AuNPs 30 s	30.88	34.36	0.154	0.645	21.31	6.65	0.861
PPy/AuNPs 45 s	29.18	24.12	0.487	0.634	39.97	6.38	0.832

## Data Availability

Not applicable.

## Supplementary Materials

The following supporting information can be downloaded at: https://www.mdpi.com/article/10.3390/molecules27185776/s1; Figure S1: Representative SEM images of PPy/AuNPs 5 s; Figure S2: Representative bright-field TEM images of (a) PPy/AuNPs 30 s, and (b) PPy/AuNPs 45 s.; Figure S3: Representative SEM images of PPy/AuNPs 15 s after AA oxidation (a) 10.000× and (b) 30.000× of magnification; Figure S4: FTIR spectra of PPy/AuNPs 15 s before (blue line) and after (brown line) the AA electrocatalysis; Table S1: Uncertainty values of the fitted parameters obtained by fitting EIS.
