# Supplementary material for: Interfacial Characterization of Polypyrrole/AuNP Composites towards Electrocatalysis of Ascorbic Acid Oxidation
Source: Molecules. 2022 Sep 7;27(18):5776. doi: 10.3390/molecules27185776 (PMC9504594; doi:10.3390/molecules27185776)
Supplement: Supplementary file 1 [file molecules-27-05776-s001.zip › molecules-1879365-supplementary.pdf]

Supplementary Information

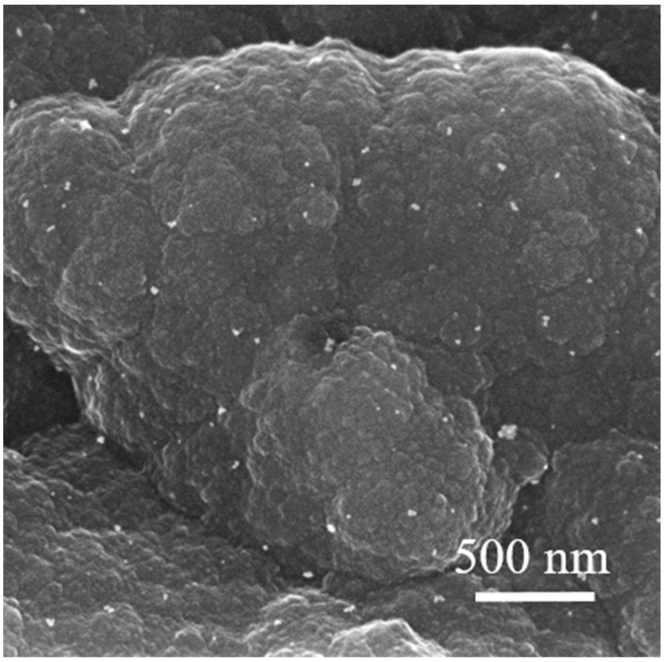

Figure S1 - Representative SEM images of modified electrode PPy/AuNPs 5s.

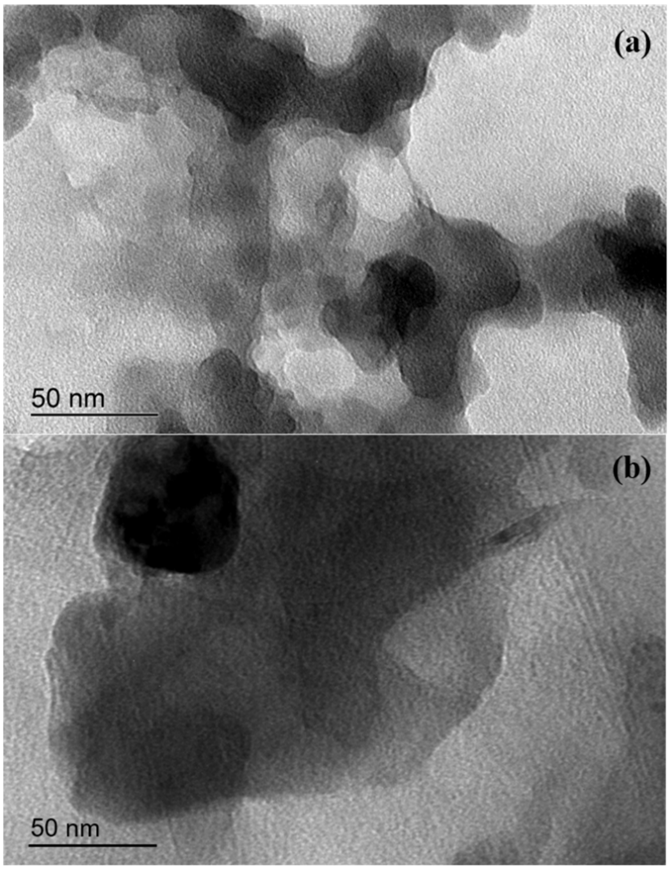

Figure S2 - Representative bright-field TEM imagens of (a) PPy/AuNPs 30s, and (b) PPy/AuNPs 45s.

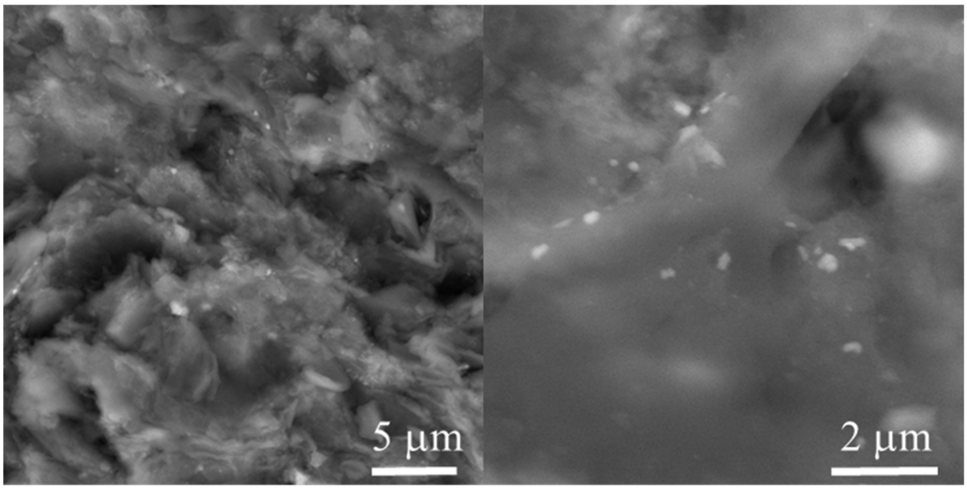

Figure S3 - Representative SEM images of PPy/AuNPs 15s after AA oxidation (a) 10.000x and (b) 30.000x of magnification.

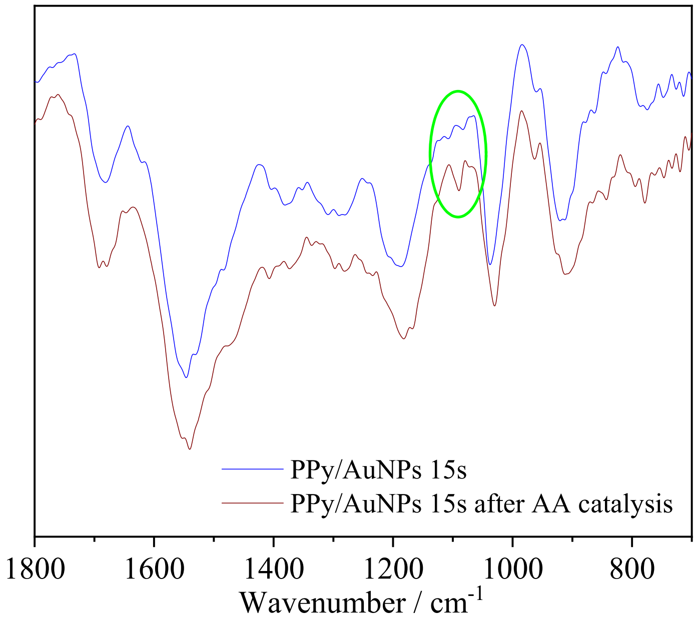

Figure S4. FTIR spectra of PPy/AuNPs 15s before (blue line) and after (brown line) the AA electrocatalysis.

Table S1. Uncertainty values of the fitted parameters obtained by fitting EIS.

Electrode	Rs/%	Rct/%	Qdl/%	ndl/%	rpol/%	qlf/%	nlf/%
PPy	0.44	1.66	4.97	1.05	9.01	0.21	0.20
PPy/AuNPs 15s	0.51	4.39	6.53	1.79	7.47	0.18	0.19
PPy/AuNPs 30s	0.44	1.18	4.84	0.99	4.84	0.17	0.15
PPy/AuNPs 45s	1.16	7.86	9.09	2.07	5.08	0.69	0.69
